# Deformation Wave Theory and Application to Optical Interferometry

**DOI:** 10.3390/ma13061363

**Published:** 2020-03-17

**Authors:** Sanichiro Yoshida, Tomohiro Sasaki

**Affiliations:** 1 Department of Chemistry and Physics, Southeastern Louisiana University, Hammond, LA 70402, USA; 2 Department of Mechanical Engineering, Niigata University, Niigata 9502181, Japan; tomodx@eng.niigata-u.ac.jp

**Keywords:** deformation and fracture of solids, Electronic Speckle-Pattern Interferometry, nondestructive testing, field theory, comprehensive theory of deformation and fracture

## Abstract

A method to diagnose the deformation status of solid objects under loading is discussed. The present method is based on a recent field theory of deformation and fracture and optical interferometry known as the Electronic Speckle-Pattern Interferometry (ESPI). Using one of the most fundamental principles of physics referred to as symmetry in physics, this field theory formulates all stages of deformation and fracture on the same theoretical basis. In accordance with the formalism, the theory has defined the criteria for different stages of deformation (linear elastic, plastic and fracturing stages) expressed by certain spatiotemporal features of the differential displacement (the displacement occurring during a small time interval). The ESPI is used to visualize the differential displacement field of a specimen as two-dimensional, full-field interferometric fringe patterns. This paper reports experimental evidence that demonstrates the usefulness of the present method. A tensile load is applied to an aluminum-alloy plate specimen at a constant pulling rate and the resultant in-plane displacement field is visualized with a two-dimensional ESPI setup. The differential displacement field is obtained at each time step and the interferometric fringe patterns are interpreted based on the criterion for each stage of deformation. It has been found that the criteria of linear elastic deformation, plastic deformation and fracturing stage are clearly observed in the corresponding fringe patterns and that the observations are consistent with the loading characteristics.

## 1. Introduction

For nondestructive evaluations of solid objects, an early detection of damage is extremely important. Those damages that lead to the final failure of an object evolve in their scale levels. Thus, the most prevailing approach is to identify the defects at the smallest level possible. Ultrasound inspection in the GHz or even higher frequency range is an example of those approaches [1,2,3]. The use of a high acoustic frequency shortens the wavelength and thereby increases the spatial resolution. However, the evolution of defects is initiated at the atomistic level. In most solids, the acoustic velocity is of the order of km/s. The wavelength corresponding to 10 GHz is still of the order of 100 nm. This is orders of magnitude greater than atomistic defects such as dislocations.

A more desirable but challenging approach is to predict the generation of damages before they evolve to a detectable size. For this approach, it is essential that the technique is based on a theory that can describe deformation and fracture on the same theoretical basis. The field theory of deformation and fracture [4,5] discussed in this paper satisfies this requirement. Based on the fundamental physical principle known as symmetry in physics [6], the theory is able to describe deformation and fracture on the same basis without relying on phenomenology. As will be discussed later in this paper, this theory yields a set of field equations that govern the dynamics of the displacement field in solids under deformation. The field equations, in turn, yield wave equations that describe deformation as wave dynamics. At different stages of deformation, different types of waves are excited under the influence of the external load. These waves represent the preferred mechanism for the material to convert the energy provided by the external load in the respective stages. In the elastic stage, the compression wave [7] converts the external energy into oscillatory energy of the particles; in the plastic stage, a transverse decaying wave [8,9,10] converts part of the external energy into the shear energy and dissipates the other part of the energy via a velocity damping mechanism; in the pre-fracture stage, a solitary wave [11] dissipates the external energy, and in the fracture stage the, the material loses all these energy conversion mechanisms and the generation of material discontinuity becomes the only possible way to balance the energy. Based on these classifications, criteria for elastic deformation, plastic deformation and fracture have been identified in a tensile analysis on a tin-plate specimen [12].

Previously, a number of experiments [10,12,13,14,15,16] were conducted to prove this field theory. In most cases, the optical interferometric technique known as Electronic Speckle-Pattern Interferometry (ESPI) [17,18] was used. Typically, the technique known as the subtraction method was used. Interferometric images of an object under deformation were formed continuously with a certain time step and the image taken at one time step was subtracted form the image taken at another time step. This procedure yielded the so-call fringe patterns where each dark fringe represents the contour of the differential displacement. Here, the word differential displacement is used to mean the displacement occurring during the time interval between the time steps when the two images involved in the subtraction were taken. By analyzing the spatial dependence of the dark fringes with various techniques, the fringe patterns were converted to the spatial dependence of the differential displacement. By repeating this procedure for multiple time steps, the wave characteristics of the differential displacement fields were observed. Consequently, the different forms of the wave characteristics in different stages of deformation were identified.

During these experiments and analyses, it has been noticed that some features of the fringe patterns uniquely appear in specific stages of deformation. This observation indicates that these features can be used to identify the respective stages of deformation. With the use of the above-mentioned field theoretical criteria of deformation stages, they can be used to diagnose the transition from one stage to another. This diagnostic method provides practical advantages to the field use of ESPI. The fringe pattern formed by the subtraction method contains the optical phase-change proportional to the differential displacement at all coordinate points. Therefore, in principle, analysis of these phase data allows us to determine the differential displacement at each coordinate point. However, in reality, the determination of the phase value at a given coordinate point is not straightforward. The phase proportional to the differential displacement, ϕ, is embedded in the optical intensity in the form of $\sin\left(\theta_{1}/2+\theta_{2}/2\right)\sin\left(\phi /2\right)$. Here, $\theta_{1}$ and $\theta_{2}$ are the optical phase in the image frames subtracted and subtracted from, respectively. Being formed by diffuse reflection from the object surface, $\theta_{1}$ and $\theta_{2}$ are random phases, hence, the term $\sin(\theta_{1}/2+\theta_{2}/2)$ is random. Due to this random term, the signal phase ϕ is undetermined from the optical intensity in the image pattern formed by subtraction. To determine the phase value at each frame absolutely, acquisition of extra frames with known phase shifts is necessary. The phase can be shifted for a known amount by either varying the optical path length from the light source to the object or varying the optical frequency. Techniques such as phase shift methods [19,20] can be used to vary the optical path length, and frequency-sweeping interferometry [21,22] can be used to vary the optical frequency. However, the formation of dark fringes by image subtraction is much simpler than any of these techniques in the optical arrangement and data acquisition procedure. In addition, the image subtraction method guarantees that the phase data is associated with the two moments from which the differential displacement is evaluated. It is possible that the deformation status changes while the optical phase is shifted. This is especially important for deformation analysis in the late stage of the plastic regime and fracturing regime because the deformation status can be changed by an abrupt phenomenon such as release of pinned dislocations.

The exception to the above situation, that the signal phase ϕ is undetermined due to the random phase term, is when ϕ is an integral multiple of 2π (ϕ=2Nπ). In this case, the sin(ϕ/2) term is zero. The dark fringes are lines connecting coordinate points where ϕ=2Nπ. Thus, we can say that along the dark fringes, the signal phase is an integral multiple of 2π without uncertainty. This fact has motivated us to use the shape of the dark fringes, instead of the phase at all coordinate points, for the identification of deformation stages and diagnosis of stage transitions. The aim of this paper is to demonstrate this idea using a tensile analysis on an aluminum alloy specimen. The above-mentioned field theoretical criteria for different deformation stages have been clearly observed as being consistent with the loading curve. This confirms the observation in ref. [12] in which the criteria were observed in a tin (Sn) specimen. It has been found that the use of a fringe shape is a quick way to diagnose the current stage of deformation without involving tedious fringe analysis.

## 2. Field Theory of Deformation and Fracture

### 2.1. Field Theory Overview

Details of the present field theory can be found elsewhere [4,5]. In short, this theory is based on two postulates. The first postulate is that a solid under plastic deformation locally obeys the law of linear elasticity. This postulate is justified by the argument that when a tensile load is removed from a plastically deformed specimen, the stress goes back to zero with the same slope as the initial linear part of the deformation on the stress–strain curve [16]. We can explain this as follows. When the deformation develops past the yield point, defects are generated causing irreversible deformation. However, local segments divided by the defects still preserve elasticity and when the load is removed, these local segments exhibit the recovery with the same slope as the initial global elasticity.

The second postulate is that all these local segments are logically connected as long as the specimen is a continuum. This postulate is supported by the concept known as the gauge (compensation) field theory in association with local symmetry [6]. In the present context, we can apply this concept to the deformation dynamics as follows. When defects divide the specimen into local segments so that the material does not obey the law of linear elasticity at the global level, we can formulaically regain the global formalism as linear elasticity by introducing a compensation field [23,24]. The discrepancy of this formulaic linear elasticity from the actual nonlinearity at the global level can be formulated by finding the dynamics that the compensation field is to obey. This procedure yields a set of field equations that we discuss in the next section.

### 2.2. Field Equations

Through application of the Lagrangian formalism [24] to the compensation field, we obtain the following set of field equations [4].

(1) $$\begin{array}{ccc} \nabla \cdot v & = & -j_{0} \end{array}$$

(2) $$\begin{array}{ccc} \nabla \times v & = & \frac{\partial \omega }{\partial t} \end{array}$$

(3) $$\begin{array}{ccc} \nabla \times \omega & = & -\frac{1}{c^{2}}\frac{\partial v}{\partial t}-J \end{array}$$

(4) $$\begin{array}{ccc} \nabla \cdot \omega & = & 0 \end{array}$$

Here, v and ω are the temporal and spatial derivatives of the displacement ξ defined by Equations (5) and (6), c is the phase velocity of the dynamics (the motion of the spatiotemporal oscillation), and $j_{0}$ and J are the temporal and spatial components of the so-called charge of symmetry. The physical meaning of $j_{0}$ and J are somewhat complicated and out of the scope of this paper. It is discussed in Chapter 5 (Section 5.4) of ref. [4]. In short, in the present context, they represent the entity that causes energy dissipation of the mechanical field.

(5) $$\begin{array}{ccc} v & = & \frac{\partial \xi }{\partial t} \end{array}$$

(6) $$\begin{array}{ccc} \omega & = & \nabla \times \xi \end{array}$$

Analysis [4] indicates that the phase velocity c can be put in the following form.

(7) $$c=\sqrt{\frac{G}{\rho }}$$

Here G is the shear modulus and ρ is the density of the material. With expression (7), field Equation (3) can be put in the following form.

(8) $$\rho \frac{\partial^{2}\xi }{\partial t^{2}}=-G\nabla \times \omega -GJ$$

The left-hand side of Equation (8) is in the form of the “acceleration” times “mass” of a unit volume. Hence, we can interpret this equation as the equation of motion for the unit volume represented by the density ρ [5,25]. Accordingly, we can interpret the two terms on the right-hand side as the external forces acting on the unit volume. Here, the first term is called the transverse elastic force as vector ω is orthogonal to the acceleration. This force is induced by shear differential displacement of neighboring blocks of material. The second term is called the longitudinal force as vector J is parallel to the acceleration. This force is induced by differential displacement or velocity. As will be discussed in the next section, when the longitudinal force is due to differential displacement, the deformation dynamics are elastic, and when it is due to differential velocity, the deformation dynamics are inelastic.

(9) $$\begin{array}{ccc} F_{\mathrm{trans}} & = & -G(\nabla \times \omega ) \end{array}$$

(10) $$\begin{array}{ccc} F_{\mathrm{long}} & = & -GJ \end{array}$$

With the negative sign in front, both forces represent resistive force.

### 2.3. Deformation Stage Criteria

#### 2.3.1. Linear Elastic Deformation

**Figure 1:**
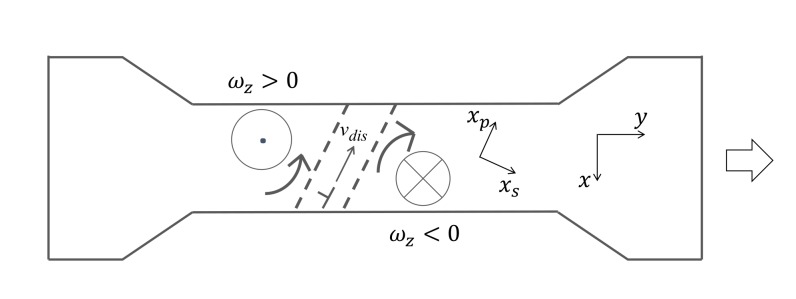
Plate specimen.

Consider in Figure 1 that a tensile force is applied to a plate specimen causing an elongation in the positive y direction. For simplicity, we discuss the case in two dimensions but the same concept applies to three dimensions. Here, the plate is in the xy-plane and the xy-system is the principal coordinate system. In accordance with Poisson’s principle, this tensile force induces only normal strains $\epsilon_{\mathrm{yy}}$ and $\epsilon_{\mathrm{xx}}$ as follows.

(11) $$\begin{array}{ccc} \epsilon_{\mathrm{yy}} & = & \frac{\partial \xi_{y}}{\partial y}=\frac{\Delta l}{l} \end{array}$$

(12) $$\begin{array}{ccc} \epsilon_{\mathrm{xx}} & = & \frac{\partial \xi_{x}}{\partial x}=\nu \frac{\partial \xi_{y}}{\partial y} \end{array}$$

where Δl is the total elongation, l is the initial length of the plate, and ν is the Poisson’s ratio. The other components of distortion tensor are null.

(13) $$\begin{array}{ccc} \epsilon_{\mathrm{xy}} & = & \frac{1}{2}\left(\frac{\partial \xi_{y}}{\partial x}+\frac{\partial \xi_{x}}{\partial y}\right)=0 \end{array}$$

(14) $$\begin{array}{ccc} \omega_{z} & = & \frac{1}{2}\left(\frac{\partial \xi_{y}}{\partial x}-\frac{\partial \xi_{x}}{\partial y}\right)=0 \end{array}$$

From Equations (13) and (14) this condition leads to the following conditions.

(15) $$\begin{array}{ccc} \frac{\partial \xi_{y}}{\partial x} & = & 0 \end{array}$$

(16) $$\begin{array}{ccc} \frac{\partial \xi_{x}}{\partial y} & = & 0 \end{array}$$

Conditions (15) and (16) indicate that both $\xi_{y}$ and $\xi_{x}$ are independent of the spatial coordinates orthogonal to them. In other words, linear elastic deformation is a uniform stretch or compression.

Under this condition consider ∇×ω

(17) $$\nabla \times \omega =\frac{\partial \omega_{z}}{\partial y}\hat{i}-\frac{\partial \omega_{z}}{\partial x}\hat{j}$$

Equation (14) guarantees that

(18) ∇×ω=0

We can see that Equation (18) is a necessary condition for linear elasticity.

With condition (18) the equation of motion (8) becomes as follows.

(19) $$\rho \frac{\partial^{2}\xi }{\partial t^{2}}=-GJ$$

The longitudinal force GJ can be identified in more than one way. Being an elastic force obeying Hooke’s law, the longitudinal force should be proportional to differential displacement. Thus, the first and most straightforward interpretation is that the elastic force is proportional to the spatial derivative of displacement vector ξ. For a unit cross sectional area, such a force can be expressed with the use of Young’s modulus E.

(20) $$F_{e}=\mathrm{AE}\frac{d\xi }{ds}$$

Here A is the area normal to the elastic force vector $F_{e}$ and dξ/ds is the normal strain with s being the coordinate variable along the axis parallel to $F_{e}$. Then, the net force acting on the unit volume represented by the density ρ is the differential force. The equation of motion becomes as follows.

(21) $$\rho \mathrm{Ads}\frac{d^{2}\xi }{dt^{2}}=dF_{e}=\frac{dF_{e}}{ds}ds=AE\frac{d^{2}\xi }{ds^{2}}\left(ds\right)$$

Equation (21) leads to the following differential equation known as the one-dimensional elastic wave equation.

(22) $$\frac{d^{2}\xi }{dt^{2}}=\frac{E}{\rho }\frac{d^{2}\xi }{ds^{2}}$$

The general solution to Equation (22) is a one-dimensional compression wave traveling at the phase velocity $\sqrt{E/\rho }$. In this case, the longitudinal elastic force $GJ_{e}$ can be identified as follows.

(23) $$GJ_{e}=-E\frac{d^{2}\xi }{ds^{2}}$$

With this force, the material resists the external force acting on the unit volume. This is the elastic resistive force discussed by conventional continuum mechanics. The longitudinal elastic force (23) can be generalized to three-dimensions by replacing the one-dimensional spatial derivative d/ds with ∇.

(24) $$GJ_{e}=-\left(\lambda +2G\right)\nabla \left(\nabla \cdot \xi \right)$$

Substituting expression (24) into the equation of motion (19) and taking the divergence of the resultant equation, we can rewrite Equation (19) as follows.

(25) $$\frac{\partial^{2}\left(\nabla \cdot \xi \right)}{\partial t^{2}}=\frac{\left(\lambda +2G\right)}{\rho }\nabla^{2}\left(\nabla \cdot \xi \right)$$

Equation (25) is the well-known compression wave equation that describes the volume expansion (∇·ξ) travels at a phase velocity $\sqrt{(\lambda +2G)/\rho }$.

Call the pairs of conditions (18) and (23), and (18) and (24), respectively, the one-dimensional and three-dimensional linear elastic criterion.

There is another possibility in linear elasticity. It is possible that dξ/ds≠0 and $d^{2}\xi /{\mathrm{ds}}^{2}=0$ (for a one-dimensional case) or ∇·ξ≠0 and ∇(∇·ξ)=0 (for a three-dimensional case). In these cases, GJ=0. The physical meaning of these cases is straightforward. These conditions of the null second-order derivatives with a finite first-order derivative mean that the stretch (or compression) is uniform. Along the axis of differentiation, the spatial change in the displacement is constant and, therefore, the elastic force proportional to the first-order derivative of displacement is the same throughout the specimen. The condition GJ=0 indicates that the net external force on a unit volume is null. Namely, all the unit volumes are in an equilibrium. Under this condition, the deformation is static and a wave is not generated.

(26) $$GJ_{e}=0$$

Call the pair of condition (18) and (26) the uniform linear elastic criterion.

#### 2.3.2. Plastic Deformation

As the deformation develops, dislocations propagate at the microscopic level. At a certain point, dislocations develop to form a macroscopic defect. The generation of a macroscopic defect divides the specimen into segments where, within each individual segment, the material preserves linear elasticity. When this happens, the linear elastic criterion is broken at the global level, but is still active at the local level within the segments. This initiates the global plastic deformation. The strain and stress tensors start to have shear components. The material still possesses elasticity and exhibits reversible stretch and compression. However, at the global level, these reversible deformations are no more in line with the external force applied to the solid object. This nonlinearity is represented by

(27) ∇×ω≠0

Condition (27) constitutes part of the plastic condition. However, it does not represent the irreversibility of deformation. The irreversibility of plastic deformation can be integrated into the formalism throught the following argument of the longitudinal force term GJ.

Take the divergence of field Equation (8) and use the mathematical identity ∇·(∇×ω)=0 and the phase velocity expression (7) to obtain the following equation.

(28) $$\frac{\partial \rho (\nabla \cdot v)}{\partial t}=-\nabla \cdot \left(GJ\right)$$

Equation (28) can be viewed as an equation of continuity where GJ is the flow of a conserved quantity ρ(∇·v). This justifies the following expression of current GJ

(29) $$GJ=W_{d}\rho \left(\nabla \cdot v\right)$$

Analysis indicates that the drift velocity vector $W_{d}$ is parallel to the particle velocity v via a material constant $\sigma_{0}$ [26].

(30) $$W_{d}=\sigma_{0}v$$

The equality (30) justifies the following expression as the plastic longitudinal force.

(31) $$GJ_{p}\equiv \sigma_{0}\rho \left(\nabla \cdot v\right)v$$

Since the direction of $GJ_{p}$ as the longitudinal force is opposite to the external force, we can interpret it as a resistive force proportional to the velocity. Call $GJ_{p}$ the plastic resistive force. Being proportional to the velocity with a negative sign, the plastic resistive force can be interpreted as a velocity damping force. It is also possible to view this force as a flow of the plastic deformation charge ρ(∇·v) with the drift velocity $W_{d}=\sigma_{0}v$. From this viewpoint, the plastic resistive force is analogous to the conduction current as a flow of an electric charge that dissipates the electric energy.

Substitution of expression (31) into the equation of motion (8) leads to the following equation.

(32) $$\rho \frac{\partial^{2}\xi }{\partial t^{2}}=-G\nabla \times \omega -\sigma_{0}\rho \left(\nabla \cdot v\right)v$$

Using Equations (5) and (6) we can rewrite Equation (32) as follows.

(33) $$\rho \frac{\partial^{2}\xi }{\partial t^{2}}+\sigma_{c}\frac{\partial \xi }{\partial t}-G\nabla^{2}\xi =-G\nabla \left(\nabla \cdot \xi \right)$$

Here $\sigma_{c}$ is defined as follows.

(34) $$\sigma_{c}=\sigma_{0}\rho \left(\nabla \cdot v\right)$$

$\sigma_{c}$ represents the degree of velocity damping effect. The right-hand side of Equation (34) indicates that increase in (∇·v) enhances the damping effect. Being the rate of volume expansion (∇·ξ), the quantity (∇·v) represents the degree of strain concentration. This observation indicates that the material becomes more energy dissipative via an increase in $\sigma_{c}$ as strain is concentrated. Later in this paper, we will observe that the interferometirc fringe pattern indeed indicates strain concentration as the deformation develops to the final fracture of the specimen.

Call the pair of conditions (27) and (31) the plastic criterion.

#### 2.3.3. Fracture

Under certain conditions, the rotational elastic force G(∇×ω) stops acting in the plastically deformed volumes. Call this phenomenon the inactivation of rotational elastic force. In this stage, the linear elasticity is not active in these volumes and hence the longitudinal elastic force is null, $GJ_{e}=0$. With both elastic forces being inactive, the differential displacement (stretch or compression represented by ∇·ξ or (∇×ω)) does not contribute to stress rise. At this point, the energy dissipative force $GJ_{p}$ is still active, and becomes the only resistive force for the unit volume. Experiments indicate that $W_{d}$ generally decreases as the deformation develops [27,28]. Consequently, being proportional to $W_{d}$, the plastic resistive force $GJ_{p}$ does not raise the stress either. When the plastic resistive force becomes inactive as well, the unit volume loses all the mechanisms to exert a resistive force against the external force.

As will be discussed later in this paper, the rotational elastic force G(∇×ω) can become inactive in any post-yield stage. If this occurs in an early post-yield stage, it is usually the case that the G(∇×ω) force is reactivated. This resumes the stress rise in proportion to ∇×ω. Normally, the process of G(∇×ω) force being inactivated and reactivated repeats, forming a zigzag pattern on the stress-strain curve [29,30]. This phenomenon is known as the serration. Whether the stress–strain curve shows a pattern of serration depends on the type of material and loading condition [28]. When a specimen exhibits a serration the inactivation of rotational elastic force takes place in certain parts of the specimen. The other part of the specimen still possesses the mechanism to exert elastic resistive force. This situation does not cause the specimen to fail (breaks). Call this condition a partial fracture condition.

When the inactivation of rotational elastic force occurs in a late post-yield stage, it often leads to the final stage of fracture. The stress monotonically decreases until the specimen fails. Experiments indicate that during this process, the drift velocity $W_{d}$ decreases in consistence with Equation (29). Eventually, $W_{d}$ becomes zero and that is when the specimen fails. Call this condition the total fracture condition.

The transition to the final failure of a specimen under the total fracture condition can be explained based on Newtonian dynamics. When the drift velocity approaches zero in Equation (29), the left-hand side GJ still represents force that is the reaction to the external force exerted by the external agent (e.g., a tensile machine). This creates a situation where the right-hand side $W_{d}\rho \left(\nabla \cdot v\right)\neq 0$ and $W_{d}=0$. The only condition to establish this situation is (∇·v)→∞. This condition indicates that particles are flowing out from a unit volume at a fast rate. Apparently, material discontinuity is generated and this is the field theoretical definition of final fracture.

Thus, the partial and total fracture criteria can be expressed as follows.

(35) $$\begin{array}{cc} \nabla \times \omega =0, & W_{d}\neq 0\;\;\;\;\;;\;\mathrm{partial}\mathrm{fracture} \end{array}$$

(36) $$\begin{array}{cc} \nabla \times \omega =0, & W_{d}=0\;\;\;\;\;;\;\mathrm{total}\mathrm{fracture} \end{array}$$

Experiments indicate that when criterion (35) or (36) is satisfied, the charge (∇·v) takes the one-dimensional form of $\left(\nabla \cdot v\right)=dv_{s}/x_{s}$ running across the specimen along the boundary of opposite rotations [31]. As illustrated in Figure 1, the charge in this form appears to be a banded structure localized at the location where the partial or total fracture occurs. Call this type of charge the developed charge [32]. Here, $x_{p}$ and $x_{s}$ are axes parallel and perpendicular to the banded structure. When the total fracture criterion is satisfied, the specimen fails at the location of the developed charge and becomes stationary [27,28]. As will be discussed later in this paper, it is believed that dislocations running along the $x_{p}$ axis are responsible for the formation of a developed charge.

## 3. Optical Method

### ESPI Fringe Pattern as Indicator of Deformation Evolution

**Figure 2:**
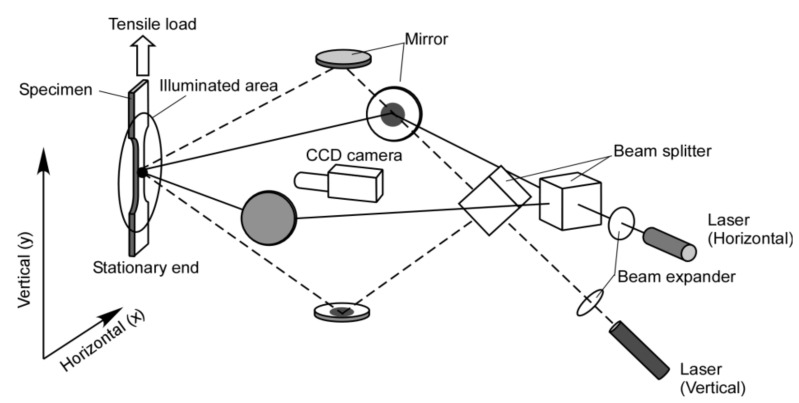
Experimental arrangement.

In this section, we present the behavior of differential displacement observed in a thin-plate specimen under tensile loading. Figure 2 illustrates the experimental arrangement. A tensile load is applied to the specimen at a constant pulling rate. The optical arrangement is as follows. Two sets of interferometers are configured where the first set is sensitive to the horizontal in-plane displacement and the second set is sensitive to the vertical in-plane displacement of all points on the specimen surface. For each interferometer, a laser source oscillating at a wavelength of 660 nm is used. The laser beam is split with a beam splitter and applied to the specimen surface. A beam expander is placed between the laser source and the beam splitter so that the laser beam illuminates the entire area of the specimen. A CCD (Charge Coupled Device) camera captures the image of the specimen at a constant time interval as the specimen is pulled by the tensile machine. To separate the image formed by the interferometer sensitive to vertical displacement from horizontal displacement, an optical switcher is used at each time step. The captured images are transferred to a computer memory where the image taken at one time step is subtracted from the image taken at another time step. The resultant subtracted image forms the so-called fringe pattern that exhibits contours of constant differential displacement occurring in the duration between the first and second time steps involved in the image subtraction. Here, the contours appear as dark lines and each dark line exhibits the differential displacement of an integral multiple of the relative phase difference between the two optical paths that form the interferometer. The fringe pattern formed by the horizontally sensitive interferometer is called the u-fringe pattern and the one formed by the vertically sensitive interferometer the v-fringe pattern hereafter.

**Figure 3:**
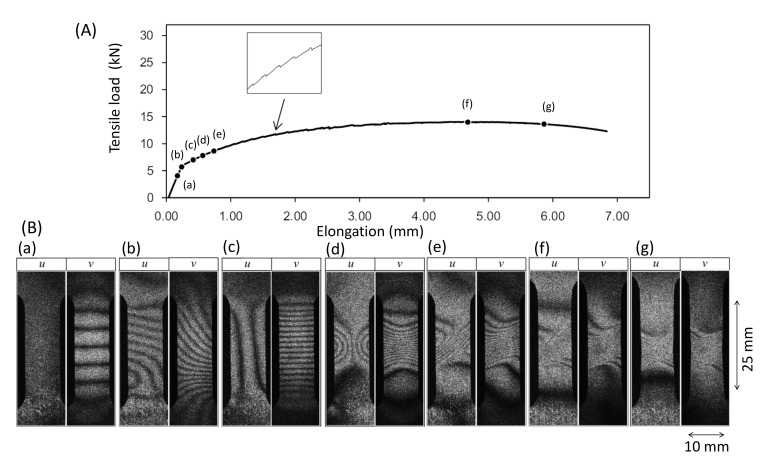
Evolution of fringe patterns observed in aluminum alloy AA7075 plate specimen. (**A**) loading curve, (**B**) fringe patterns.

Figure 3 shows a pair of fringe patterns obtained with a two-dimensional in-plane sensitive ESPI setup for a tensile experiment [33]. The specimen is a plate of 10 mm × 25 mm effective area with 5 mm thickness. The material is an industrial Al–Zn–Mg–Cu alloy AA 7075 with the following heat treatment. Before being cut to the 10 mm × 25 mm specimen, the AA7075 plate of 5 mm thickness was solid-solution-treated and hardened up to the peak hardness by non-scale precipitates. Subsequently, the plate was over-aged at 400 ${}^{\circ }$C for 30 min so that the matrix was softened through coarsening of the precipitates. By these treatments, the alloy is considered to be macroscopically homogeneous.

Figure 3A is the loading curve of the tensile experiment. Figure 3B are the u- and v-fringe patterns formed at several stages during the tensile experiment marked on the loading curve as (a)–(g). Here, (a)–(g) put above each pair of the fringe patterns indicate the stages.

These fringe patterns exhibit the following features at each stage.

1. Point (a); Pre-Yield stage
  Prior to the yield point the dark fringes observed in the *u* and *v*- fringe patterns are linear.
2. Point (b); Yield point
  At the yield point the fringes become curved. The fringe pattern is asymmetric for both the *u*-pattern and *v*-pattern.
3. Point (c); Post-Yield 1 stage
  Shortly (0.6 % in strain) after the yield point the fringes become symmetric. Here, the dark fringes in the *u*-pattern and *v*-pattern are approximately symmetric vertically and horizontally symmetric about the central horizontal line. Dark fringes are slightly curved.
4. Point (d); Post-Yield 2 stage
  Similar to the Post-Yield 1 stage, dark fringes in the *u*-pattern and *v*-pattern exhibit the symmetric feature. However, as compared with the Post-Yield 1 stage the dark fringes are much more curved. In addition, they are concentrated near the vertical center of the specimen (strain concentration).
5. Point (e); Post-Yield 3 stage
  The dark fringes in the *u*- and *v*-patterns lose the horizontal and vertical symmetry. Instead, they are somewhat symmetric around the slant, banded-region running from the lower left to the upper right, as seen in Figure 3, in which parallel, nearly linear dark fringes, are formed. Unlike the earlier stages, the *u*-pattern and *v*-pattern are similar to each other. The degree of strain concentration is similar to Point (d).
6. Point (f); Post-Yield 4 stage
  The slant symmetric feature of the fringe pattern is similar to Point (e). The similarity of the *u*- and *v*-patterns is clearer than Point (e). The degree of strain concentration is similar to Point (e).
7. Point (g); Post-Yield 5 stage
  The overall fringe pattern is similar to Point (e) while the degree of strain concentration is higher. The *u*- and *v*-patterns are nearly identical to each other.

## 4. Discussions

In this section, we interpret the fringe patterns formed by the ESPI experiment based on the field theory. After considering some general features of the differential displacement vector components when they are expressed as second or first-order polynomial functions of x and y, we discuss the actual fringe patterns seen in Figure 3 accordingly.

### Basic Fringe Patterns

The dark fringes observed in Figure 3 can be expressed as a second-order polynomial functions of x and y as follows. Here, x and y are the coordinate variables set up in the plane of the fringe image. Their orientations are the same as Figure 2.

(37) $$\begin{array}{ccc} u(x,y) & = & \Delta \xi_{x}=a_{2}\left(y-y_{0}\right)x^{2}+a_{1}\left(x-x_{0}\right)+b_{2}\left(x-x_{0}\right)y^{2}+b_{1}\left(y-y_{0}\right) \end{array}$$

$$\begin{array}{ccc} & = & a_{1}\left(x-x_{0}\right)+b_{2}\left(x-x_{0}\right)y^{2}+b_{1}\left(y-y_{0}\right) \end{array}$$

(38) $$\begin{array}{ccc} v(x,y) & = & \Delta \xi_{y}=c_{2}\left(y-y_{0}\right)x^{2}+c_{1}\left(x-x_{0}\right)+d_{1}\left(y-y_{0}\right)+d_{2}\left(x-x_{0}\right)y^{2} \end{array}$$

$$\begin{array}{ccc} & = & c_{2}\left(y-y_{0}\right)x^{2}+c_{1}\left(x-x_{0}\right)+d_{1}\left(y-y_{0}\right) \end{array}$$

Here $a_{2}\neq 0$ represents the situation where the dark fringes are not equally distanced in a ***u***-fringe pattern and $d_{2}\neq 0$ represents the same situation for a ***v***-fringe pattern. For fringe patterns seen in Figure 3, we can assume that $a_{2}=d_{2}=0$. The second lines for Equations (37) and (38) are the expressions with these assumptions taken into account. Linear fringes such as those seen at stage (a) in Figure 3 are interpreted as the case where the corresponding second order coefficient $b_{2}$ or $c_{2}$ is zero.

**Figure 4:**
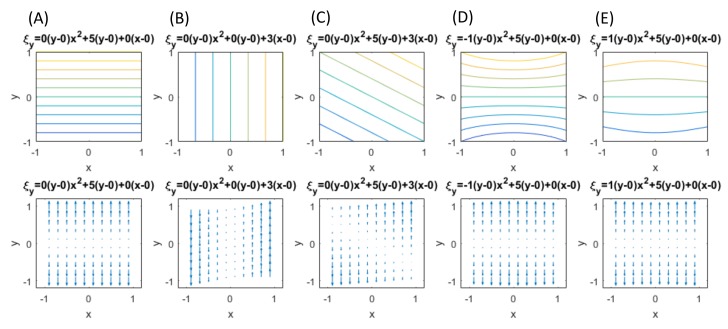
Basic types of *v* fringes. (**A**–**E**) are typical patterns obsered in tensile experiment with ESPI.

Figure 4 illustrates several basic patterns generally observed in the vertical fringes formed in the same type of tensile experiments as the present one. The upper illustration represents contours of $v\left(x,y\right)=\Delta \xi_{y}$ simulating the dark fringes. The lower illustration is a quiver plot showing the spatial variation of $\Delta \xi_{y}$. The second-order polynomial expressions above each vertical pair of the illustration indicate Equation (38) with hypothetical coefficients for the polynomial terms. Each pattern can be briefly characterized as follows.

(A) The v-fringes are linear functions of y, independent of x. In Equation (38) this means $c_{1}=c_{2}=0$. This is a case of uniform elastic deformation expressed in the coordinate system of the principal axes. The quiver plot indicates uniform stretch. In the language of the present field theory, the stretch is global.
(B) The v-fringes are linear functions of x, independent of y. In Equation (38) $c_{2}=d_{1}=0$. The quiver plot indicates rigid-body rotation expressed in the coordinate system of the principal axes. The entire specimen undergoes the same rotation. In the language of the present field theory, the rotation is global.
(C) The v-fringes are linear functions of x and y. In Equation (38) $c_{2}=0$. The quiver plot indicates a combination of uniform stretch and rigid-body rotation. The entire specimen undergoes the same rotation (the rotation is global).
(D) The v-fringes are quadratic functions of y, independent of x, with a negative coefficient for the second-order term. The parabolic fringe pattern bulges inward (called a “concave” parabolic pattern). The quiver plot indicates the following local rotations; the first and third quadrants undergo clockwise rotation and the second and fourth quadrants counterclockwise rotation.
(E) The v-fringes are quadratic functions of y, independent of x, with a positive coefficient for the second-order term. The parabolic fringe pattern bulges outward (called a “convex” parabolic pattern). The quiver plot indicates the following local rotations; the first and third quadrants undergo counterclockwise rotation and the second and fourth quadrants clockwise rotation.

With expressions (37) and (38), the shear strain (13) and rotation (14) in the xy-plain, the volume expansion ∇·ξ, the longitudinal elastic force (24) and transverse elastic force (9) can be written as follows.

(39) $$\begin{array}{ccc} \epsilon_{\mathrm{xy}} & = & \frac{1}{2}\left(2b_{2}\left(x-x_{0}\right)y+b_{1}+2c_{2}\left(y-y_{0}\right)x+c_{1}\right) \end{array}$$

(40) $$\begin{array}{ccc} \omega_{z} & = & \frac{1}{2}\left(2c_{2}\left(y-y_{0}\right)x+c_{1}-2b_{2}\left(x-x_{0}\right)y-b_{1}\right) \end{array}$$

(41) $$\begin{array}{ccc} \nabla \cdot \xi & = & a_{1}+b_{2}y^{2}+c_{2}x^{2}+d_{1} \end{array}$$

(42) $$\begin{array}{ccc} GJ_{e} & = & -\left(\lambda +2G\right)\nabla \left(\nabla \cdot \xi \right)=-\left(\lambda +2G\right)\left(2c_{2}x\;\hat{i}+2b_{2}y\;\hat{j}\right) \end{array}$$

(43) $$\begin{array}{ccc} G\nabla \times \omega & = & G\left(c_{2}x-b_{2}\left(x-x_{0}\right)\right)\hat{i}+G\left(b_{2}y-c_{2}\left(y-y_{0}\right)\right)\hat{j} \end{array}$$

Table 1 lists six typical combinations of the u- and v-fringe patterns. The second and third columns indicate the classification of the fringe patters as follows; the fringes are linear (L) or nonlinear (nL), and running horizontally (H), vertically (V) or slantingly (S). The column labeled “Coefficients” indicates the coefficients of the polynomial expressions used to express the shape of the u- and v-fringes. The column labeled “Remarks” indicates examples of each case. Expressions (37) and (38) together with Equations (39)–(41) indicate it possible to relate the shape of the dark fringes to shear strain $\epsilon_{\mathrm{xy}}$, rotation $\omega_{z}$ and the volume expansion ∇·ξ. Table 2 shows the expression of $\epsilon_{\mathrm{xy}}$, $\omega_{z}$ and ∇·ξ for each case listed in Table 1.

Table 1 and Table 2 indicate the following behaviors of deformation for each case.

Case (1) This is a linear elastic deformation such as the one observed in Figure 3 point (a). Obeying the Poisson’s effect, the specimen exhibits inward horizontal displacement (towards the vertical center of the specimen) and upward vertical displacement in response to the vertical tensile load. The ratio of the horizontal normal strain to the vertical normal strain is equal to the Poisson’s ratio of the material. Non-zero volume expansion ∇·ξ≠0 indicates that the specimen experiences stretch or compression. Constant volume expansion $\nabla \cdot \xi =a_{1}+d_{1}$ indicates that the stretch or compression is uniform.
Case (2) This pattern is seen in the upper part of Figure 3 point (b). Zero volume expansion ∇·ξ=0 indicates that the specimen is not deformed. It follows that shear strain $\epsilon_{\mathrm{xy}}=0$ and hence the displacement field exhibits rigid-body rotation represented by $\omega_{z}=c_{1}$. This pattern is commonly observed in the pre-fracture stage in tensile experiments where shear strain is concentrated in a small area above and below which the specimen undergoes rigid-body rotations [31].
Case (3) This is a combination of Case (1) and Case (2). The ***u***- and ***v***-fringes are slant because both have x and y dependence. With the second order coefficient zero, the non-linear longitudinal elastic force is still zero. Shear strain and rotation are bodily (independent of the spatial coordinates). This indicates that the global horizontal and vertical coordinate axes (x and y) do not represent the principal axes. In the context of the tensile experiment, this pattern normally indicates that the specimen undergoes concentrated deformation outside the area of view (such as the shoulder of a dog-bone shape specimen) which induces misalignment between the tensile force exerted by the test machine and the vertical axis. The non-zero volume expansion indicates that the specimen undergoes deformation. The constant volume expansion indicates that the deformation is uniform in the area of view. Unlike Case (1), the shear strain is non-zero because it is expressed with non-principal axes.
Case (4) The u-fringes are parabolic and the v-fringes are horizontally straight. This pattern is seen in point (c) in Figure 3. Shear strain and rotation are spatial coordinate-dependent, and the non-linear longitudinal elastic force only has a vertical (y)-component.
Case (5) Both the u- and v-fringes are parabolic where the u-fringes are running vertically (independent of x) and the v-fringes are horizontal (independent of y). This pattern is seen in point (d) in Figure 3. The shear strain and rotation are spatial-coordinate-dependent and the non-linear longitudinal elastic force has x- and y-components.
Case (6) This is the most general case for the u(x,y) and v(x,y) expressions (37) and (38). The fringe images at point (e) in Figure 3 exhibit this pattern. The u- and v-fringes are nonlinear and the linear terms depend on x and y.

Table 3 summarizes the above observations.

The above arguments regarding the fringe patterns can be summarized as follows.

1. Linear fringes: If u-fringes are vertical and v-fringes are horizontal, the deformation is linear and elastic. The material exhibits normal strains only (when expressed with the principal axes). If u-fringes are horizontal and v-fringes are vertical or both fringes are slant, the deformation contains shear strain or rotation. In any case, the non-linear longitudinal elastic force is null.
2. Parabolic fringes: When fringes are parabolic, the deformation contains shear strain and/or rotation, and the non-linear longitudinal elastic force is non-zero. The parabolic fringe pattern can be convex or concave, which represents local rotational displacement patters, as will be described below.

Based on the basic patterns shown in Figure 4, we can interpret the behavior of the differential displacement observed in Figure 3B in association with transition frm deformation to fracture. The following arguments can be made.

**Figure 5:**
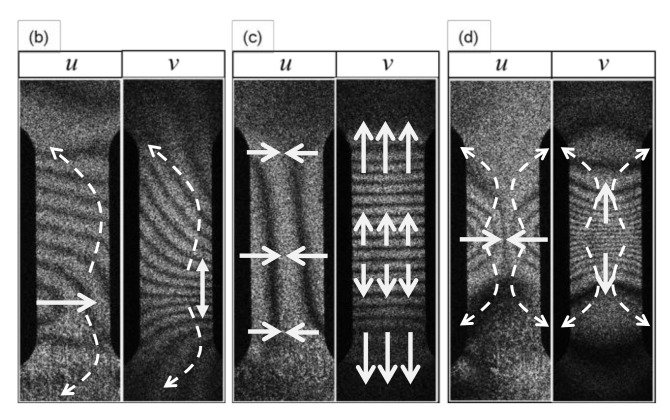
Interpretation of fringe pattern evolution. (b–d) here match (b–d) in Figure 3 and the texts under Figure 3.

**Figure 6:**
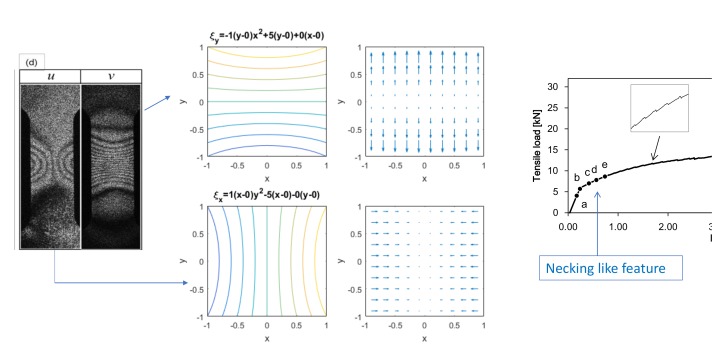
Differential displacement fields at Post-Yield 2 stage represented by fringe pattern (d) in Figure 5.

**Figure 7:**
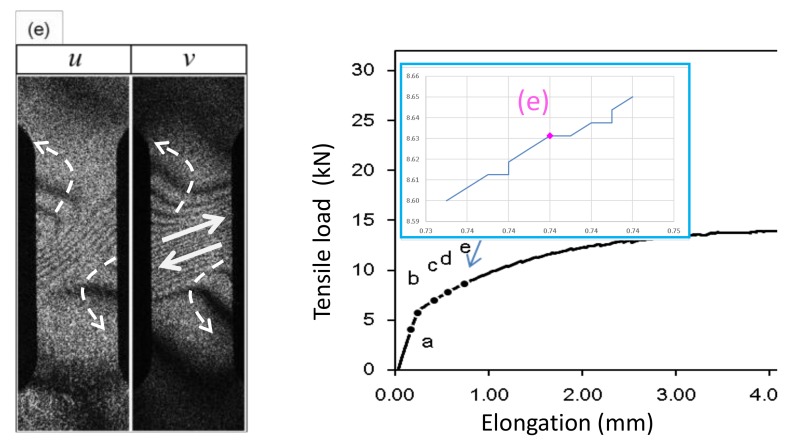
Developed shear stress at Post-Yield 3 stage represented by fringe pattern (e) in Figure 3 and subsequent loading behavior.

1. Point (b); Yield point
  Figure 5b indicates the nature of differential displacement of this stage. The ***u***- and ***v***-fringe patterns exhibit the concave type discussed in Figure 4d (The ***v***-fringe pattern is represented by the left half of Figure 4d. The ***u***-fringe pattern is represented by the left half of Figure 4d after rotated clockwise by 90${}^{\circ }$ so that it represents $\Delta \xi_{x}$.). The ***u***-fringe pattern indicates that the region approximately a quarter from the bottom end of the specimen divides the differential displacement pattern such that the top three quarters exhibits counterclockwise rotation ( $\omega_{z}>0$) and the bottom quarter exhibits clockwise rotation ( $\omega_{z}<0$). The fringes away from this dividing regions are linear function of x and y, clearly satisfying the elastic criterion (18). The tilting behavior of the linear fringes indicates local bodily rotation. The v-fringe pattern shows the same rotational behaviors. As the entire specimen, the rotational behavior that $\omega_{z}>0$ above the dividing region and $\omega_{z}<0$ below the dividing region indicates that the plastic deformation criterion (27) is satisfied. In other words, this pair of u- and v-fringe patterns literally exhibit the locally linear elastic and globally nonlinear plastic situation discussed above. The differential rotational behavior makes the dividing region undergo large rightward displacement as the arrow in Figure 5b indicates.
2. Point (c); Post-Yield 1 stage
  The ***u***- and ***v***-fringes exhibit the concave features discussed in Figure 4d. This interpretation allows us to draw the differential displacement as indicated in Figure 5c. The curvature of the concave fringes is low, indicating that the nonlinearity is low.
3. Point (d); Post-Yield 2 stage
  The concave feature in the u- and v-fringes is the same as stage (c) but the curvature is much greater and the fringes are much more concentrated towards the vertical center of the specimen. Around the center of the specimen, the top-right and bottom-left exhibit clockwise rotation ($\omega_{z}<0$) and the top-left and bottom-right exhibit counterclockwise rotation ($\omega_{z}>0$). It is clear that the plastic criterion ∇×ω≠0 (27) is satisfied. Figure 6 illustrates the spatial pattern of the differential displacement components $u=\Delta \xi_{x}$ and $v=\Delta \xi_{y}$ explicitly. Notice that the behavior in the u-field indicates that the so-called necking starts at this stage of deformation.
4. Point (e); Post-Yield 3 stage
  The u- and v-fringe patterns at this stage exhibit three distinctive regions. The middle region is characterized by approximately linear, slant dark fringes. The upper and lower regions are characterized by concave-curved fringes. Unlike the concave fringes observed at Point (d), however, the patterns are asymmetric. In the upper region, the curvature is much greater on the left half than the right half. Conversely, in the lower region, the right half exhibits a much greater curvature than the left half. Based on the argument made for Point (d) (see Figure 5d), we can say that the top left and bottom right regions undergo counterclockwise rotations ($\omega_{z}>0$). As indicated in Figure 7 these rotations induce shear stress on the right and left sides of the specimen. The linear slant fringes in the middle region leads to the condition ∇×ω=0 (because the linearity indicates a spatially independent ω), and hence satisfies partial fracture condition (35). Indeed, the closed-up loading curve inserted in Figure 7 indicates that immediately after point (e) the stress stops rising. It is likely that the shear stress causes the partial fracture condition.
  This establishment of partial fracture condition in conjunction with the temporal behavior of the stress can be explained from the viewpoint of dislocation theory. The shear stress induced by the rotation pair pushes dislocations toward a side of the specimen along the boundary of the rotations. The material exerts resistive force to the dislocations by various mechanisms, such as dislocation pinning by impurities [34]. This force causes the stress rise as observed in the loading curve. Overcoming the resistive force, the dislocations keep propagating. Once the propagation reaches a side of the specimen, the exertion of resistive force ceases. Consequently, the stress drops. At the same time, the upper and lower part recoil induces rotations respectively. Since the angular momentum must be conserved (because there is no external torque), the rotations are opposite in direction. The same process repeats at the boundary of another pair of rotations where high shear stress is generated.
  This type of repetitive stress rise and drop is known as the serration. A number of previous studies [27,28,29,30] revealed clear correlations between serration and rotation-like fringe patterns. When a uniform stretch is hindered at a point where the dislocation is pinned, the stretch over the unpinned portions causes rotation because the deformation is hinged at the point of pinning. Previous studies [10,27] indicate that the slant, linear fringe patterns observed in association with the rotational behavior of deformation move at a velocity similar to the rotational wave. This makes sense because, as mentioned above, the formation of shear stress is caused by the rotational mode displacement. It is likely that the high shear stress is developed at the location where the rotational wave has a peak. It is interesting to note that pure aluminum specimens normally do not show a clear serration behavior and the post-yield parabolic fringe patterns are usually not slant. This can be explained as follows. When the material is free of impurity, the dislocations are not pinned and, therefore, the material undergoes deformation without the type of rotations that exhibit slant, non-linear fringes. The formation of the shear stress along with the associated stress drop and the explanation based on dislocation theory is a subject of our future study.
5. Point (f); Post-Yield 4 stage
  This point is characterized as the maximum stress point on the loading curve Figure 3A. The similarity in the overall fringe pattern to Point (e) indicates that the developed shear deformation along the boundary of the counterclockwise rotation pair is still active. The fact that the stress starts to decrease from this point on indicates reduction in the elastic modulus. The propagation of dislocations associated with the serration is responsible for the reduction in the elastic modulus. The enhancement in the similarity between the ***u***- and ***v***-fringe patterns indicates that this is the onset of the transition to the final fracture (see the discussion for Point (g) in the next paragraph).
6. Point (g); Post-Yield 5 stage
  The higher degree in the strain concentration as compared with Point (g) indicates that a developed, localized charge is being formed. The similarity between the ***u***- and ***v***-fringes leads to an interesting argument. Previous experiments [12,27] commonly indicate that at the fracture the ***u***- and ***v***-fringe patterns are identical in shape where the dark fringes are linear. The linearity of the dark fringes in this context makes total sense as linear differential displacement makes ∇×ω=0 and this condition constitutes the fracture criterion (36). Thus, the following argument can be made. Assume that the horizontal and vertical differential displacements are expressed by the same function as
  (44) $$u\left(x,y\right)=v\left(x,y\right)=f\left(x,y\right)=a\left(x-x_{0}\right)+b\left(y-y_{0}\right)$$
  Substitution of Equation (44) into Equations (39) and (41) leads to the following equality.
  (45) $$\begin{array}{ccc} \epsilon_{\mathrm{xy}} & = & \frac{1}{2}\left(\frac{\partial v}{\partial x}+\frac{\partial u}{\partial y}\right)=\frac{1}{2}\left(a+b\right) \end{array}$$
  (46) $$\begin{array}{ccc} \nabla \cdot \xi & = & \frac{\partial u}{\partial x}+\frac{\partial v}{\partial y}=a+b \end{array}$$
  Equations (45) and (46) indicate $\epsilon_{\mathrm{xy}}\propto \nabla \cdot \xi$. Remembering that the plastic deformation charge ∇·v is the temporal derivative of ∇·ξ we can argue that in the fracturing stage the charge is characterized as shear. This view is consistent with the view by Panin [35] that the plastic deformation is shear instability and the fracture is the final stage of plastic deformation. In addition, u and v are the x and y components of the differential displacement vector Δξ. The fact that the x and y components are equal to each other means that the vector Δξ is oriented 45${}^{\circ }$ to the tensile direction (y-axis). This is the direction of the maximum shear stress and it is widely known that fracture occurs in the direction of the maximum shear stress.

## 5. Concluding Remarks

Tensile deformation of a thin plate specimen has been discussed based on the field theory in conjunction with fringe patterns formed with ESPI. The evolution from the initial elastic deformation to a late stage of plastic deformation has been analyzed from the view point of the field theoretical criterion for each stage. It has been found that the fringe image reveals the criteria as specific patterns of the dark fringes. The main results are as follows.

1. If the dark fringes observed in the differential displacement component parallel to the tensile axis are equidistant, linear parallel running perpendicularly to the tensile force, the deformation is linear and elastic. Under this condition, the dark fringes of the differential displacement component perpendicular to the tensile axis are equidistant, linear, and running parallel to the tensile force. The numbers of fringes observed in the two components are determined by the Poisson’s ratio of the material. The shear strain $\epsilon_{\mathrm{xy}}$ and rotation $\omega_{z}$ are zero when expressed in the principal coordinate system.
2. If the dark fringes are slanted to the tensile axis, it indicates that $\epsilon_{\mathrm{xy}}\neq 0$ and/or $\omega_{z}\neq 0$ meaning that the deformation is plastic. It is likely that dark fringes are curved indicating that the plastic deformation criterion is established, ∇×ω≠0. It is possible that curved fringes are not in the view of the fringe image. In that case, dark fringes appear to be slant and linear.
3. As the deformation further develops, the dark fringes tend to be concentrated at a certain location of the specimen. It is possible that the concentrated dark fringes are approximately linear, running at a slanted angle to the tensile axis and that above and below these slanted regions, the fringes are curved. In that case, if the upper and lower curved fringes represent $\omega_{z}$ of the same sign, it means that the material experiences concentrated shear strain in the slanted region. It is likely that the partial fracture criterion is satisfied and that the stress is about to drop.
4. In the late stage of plastic deformation, when the loading curve has passed the maximum stress, the fringe patterns observed in the perpendicular and parallel components of the differential displacement start to become similar to each other. This is an indication that the deformation enters the final stage toward the total fracture. The fringe patterns in the perpendicular and parallel components of the differential displacement become identical on the final fracture.

Although the present study focused on the analysis of an aluminum alloy specimen, similar evolutions of fringe patterns with the development of tensile deformation have recently been observed in steel and other metals. Preliminary analysis indicates that the observed fringe patterns are consistent with the field theoretical criteria discussed in this paper. Further analyses of these results are currently being conducted.

## Figures and Tables

**Table 1:** ***u***- and ***v***-fringe shapes and corresponding coefficients of polynomial expressions.

Case	*u*-Fringes	*v*-Fringes	Coefficients	Remarks
(1)	L/V	L/H	$b_{2}=b_{1}=c_{2}=c_{1}=0$, $a_{1}\neq 0,d_{1}\neq 0$	Observed in Figure 3 point (a)
(2)	L/H	L/V	$b_{2}=a_{1}=c_{2}=d_{1}=0$, $b_{1}\neq 0,c_{1}\neq 0$	v-fringes shown in Figure 4b
(3)	L/S	L/S	$b_{2}=c_{2}=0$, $a_{1}\neq 0,b_{1}\neq 0,c_{1}\neq 0,d_{1}\neq 0$	Observed in Figure 3 point (b)
(4)	nL/V	L/H	$b_{1}=c_{2}=c_{1}=0$, $b_{2}\neq 0,a_{1}\neq 0,d_{1}\neq 0$	Observed in Figure 3 point (c)
(5)	nL/V	nL/H	$b_{1}=c_{1}=0$, $b_{2}\neq 0,c_{2}\neq 0,a_{1}\neq 0,d_{1}\neq 0$	Observed in Figure 3 point (d)
(6)	nL/S	nL/S	$b_{2}\neq 0,c_{2}\neq 0,a_{1}\neq 0,b_{1}\neq 0,c_{1}\neq 0,d_{1}\neq 0$	Observed in Figure 3 point (e)

**Table 2:** ***u***- and ***v***-fringe shapes and corresponding shear, rotation and volume expansion.

Case	$\epsilon_{\mathrm{xy}}$	$\omega_{z}$	∇·ξ
(1)	0	0	$a_{1}+d_{1}$
(2)	$\frac{1}{2}\left(b_{1}+c_{1}\right)=0$	$\frac{1}{2}\left(-b_{1}+c_{1}\right)=c_{1}$	0
(3)	$\frac{1}{2}\left(b_{1}+c_{1}\right)$	$\frac{1}{2}\left(-b_{1}+c_{1}\right)$	$a_{1}+d_{1}$
(4)	$b_{2}\left(x-x_{0}\right)y$	$-b_{2}\left(x-x_{0}\right)y$	$a_{1}+d_{1}+b_{2}y^{2}$
(5)	$b_{2}\left(x-x_{0}\right)y+c_{2}\left(y-y_{0}\right)x$	$-b_{2}\left(x-x_{0}\right)y+c_{2}\left(y-y_{0}\right)x$	$a_{1}+d_{1}+b_{2}y^{2}+c_{2}x^{2}$
(6)	$\frac{1}{2}\left(2b_{2}\left(x-x_{0}\right)y+b_{1}+2c_{2}\left(y-y_{0}\right)x+c_{1}\right)$	$\frac{1}{2}\left(2c_{2}\left(y-y_{0}\right)x+c_{1}-2b_{2}\left(x-x_{0}\right)y-b_{1}\right)$	$a_{1}+d_{1}+b_{2}y^{2}+c_{2}x^{2}$

**Table 3:** ***u***- and ***v***-fringe shapes and corresponding deformation stage.

Case	G(∇×ω)	GJ	Deformation Stage Criteria
(1)	0	0	uniform elastic deformation (principal axes)
(2)	0	0	rigid-body rotation
(3)	0	0	uniform elastic deformation (non-principal axes)
(4)	$-{\mathrm{Gb}}_{2}\left(x-x_{0}\right)\;\hat{i}$	$-2\left(\lambda +2G\right)b_{2}y\;\hat{j}$	plastic deformation
(5)	$-G\left(b_{2}\left(x-x_{0}\right)\;\hat{i}+c_{2}\left(y-y_{0}\right)\;\hat{j}\right)$	$-2\left(\lambda +2G\right)\left(c_{2}x\;\hat{i}+b_{2}y\;\hat{j}\right)$	plastic deformation
(6)	$-G\left(b_{2}\left(x-x_{0}\right)\;\hat{i}+c_{2}\left(y-y_{0}\right)\;\hat{j}\right)$	$-2\left(\lambda +2G\right)\left(c_{2}x\;\hat{i}+b_{2}y\;\hat{j}\right)$	plastic deformation
